# Missing in action: Species competition is a neglected predictor variable in species distribution modelling

**DOI:** 10.1371/journal.pone.0181088

**Published:** 2017-07-14

**Authors:** Kudzai Shaun Mpakairi, Henry Ndaimani, Paradzayi Tagwireyi, Tawanda Winmore Gara, Mark Zvidzai, Daphine Madhlamoto

**Affiliations:** 1 Department of Geography and Environmental Science, University of Zimbabwe, Harare, Zimbabwe; 2 Faculty of Geo-Information Science and Earth Observation (ITC), University of Twente, Enschede, The Netherlands; 3 Zimbabwe Parks and Wildlife Management Authority, Gonarezhou National Park, Chiredzi, Zimbabwe; University of Minnesota, UNITED STATES

## Abstract

The central role of species competition in shaping community structure in ecosystems is well appreciated amongst ecologists. However species competition is a consistently missing variable in Species Distribution Modelling (SDM). This study presents results of our attempt to incorporate species competition in SDMs. We used a suit of predictor variables including Soil Adjusted Vegetation Index (SAVI), as well as distance from roads, settlements and water, fire frequency and distance from the nearest herbivore sighting (of selected herbivores) to model individual habitat preferences of five grazer species (buffalo, warthog, waterbuck, wildebeest and zebra) with the Ensemble SDM algorithm for Gonarezhou National Park, Zimbabwe. Our results showed that distance from the nearest animal sighting (a proxy for competition among grazers) was the best predictor of the potential distribution of buffalo, wildebeest and zebra but the second best predictor for warthog and waterbuck. Our findings provide evidence to that competition is an important predictor of grazer species’ potential distribution. These findings suggest that species distribution modelling that neglects species competition may be inadequate in explaining the potential distribution of species. Therefore our findings encourage the inclusion of competition in SDM as well as potentially igniting discussions that may lead to improving the predictive power of future SDM efforts.

## Introduction

Ecologists appreciate the central role of competition among species in shaping community structure within ecosystems [1, 2]. Species compete for a suit of resources including food [3, 4]; water [5, 6] and space [7, 8]. Heightened competition among species may lead to community reorganization as the weaker competitor is often excluded, replaced and possibly suffers local extinction [9, 10]. However, some species adapt through niche differentiation [11] by avoiding patches where their access to resources is compromised with competition from individuals of the same species (intraspecific competition) or those from another species (interspecific competition) [12]. It is also noteworthy that for some species, the effect of competition on community structure remains largely negligible [13]. These mechanisms put together, help to explain why species select or avoid particular patches. It is for this reason that explaining the geographic distribution of species without using competition as a predictor may not be adequate. However the inclusion of species competition in SDMs remains a grey area [6].

The reasons for the exclusion of competition as a predictor variable in SDMs are many including challenges in rasterizing competition. SDMs require continuous surfaces (e.g., raster data) as predictor variables [14, 15] but currently, there lacks a formally documented method for rasterizing species competition despite the documented central role of competition in explaining species distribution. Some studies have estimated competition from point observations made in the field [16, 17]. However, to the best of our knowledge, past species distribution modelling attempts (using SDMs) did not use competition as a predictor variable.

In this study, we hypothesized that competition (estimated by distance from the nearest animal sighting) is a key predictor of the potential geographic distribution of selected grazers. We propose that physical separation of two species in a landscape is a strategy adopted to avoid direct competition for resources such as forage and water. Thus the further away the species are located in the landscape, the lesser the competition and the closer they are, the more the competition. To test this hypothesis we determined the relative contribution of competition to predicting the potential distribution of selected grazers as well as the response of selected individual grazers to competition. To achieve this, we used the Ensemble SDM [18] with distance from the nearest sighting together with five other key traditional predictor variables to predict the potential geographic distribution of: buffalo (*Syncerus caffer*); warthog (*Phacochoerus africanus*); waterbuck (*Kobus ellipsiprymnus*); wildebeest (*Connochaetes taurinus*), and, zebra (*Equus quagga*) in Gonarezhou National Park, Zimbabwe. We anticipate that the results of this research will find use in ecological applications that seek to predict the potential distribution of species by encouraging the incorporation of species competition, a key driver of species distribution that has been neglected in past SDMs.

## Materials and methods

### Study site

**Fig 1:**
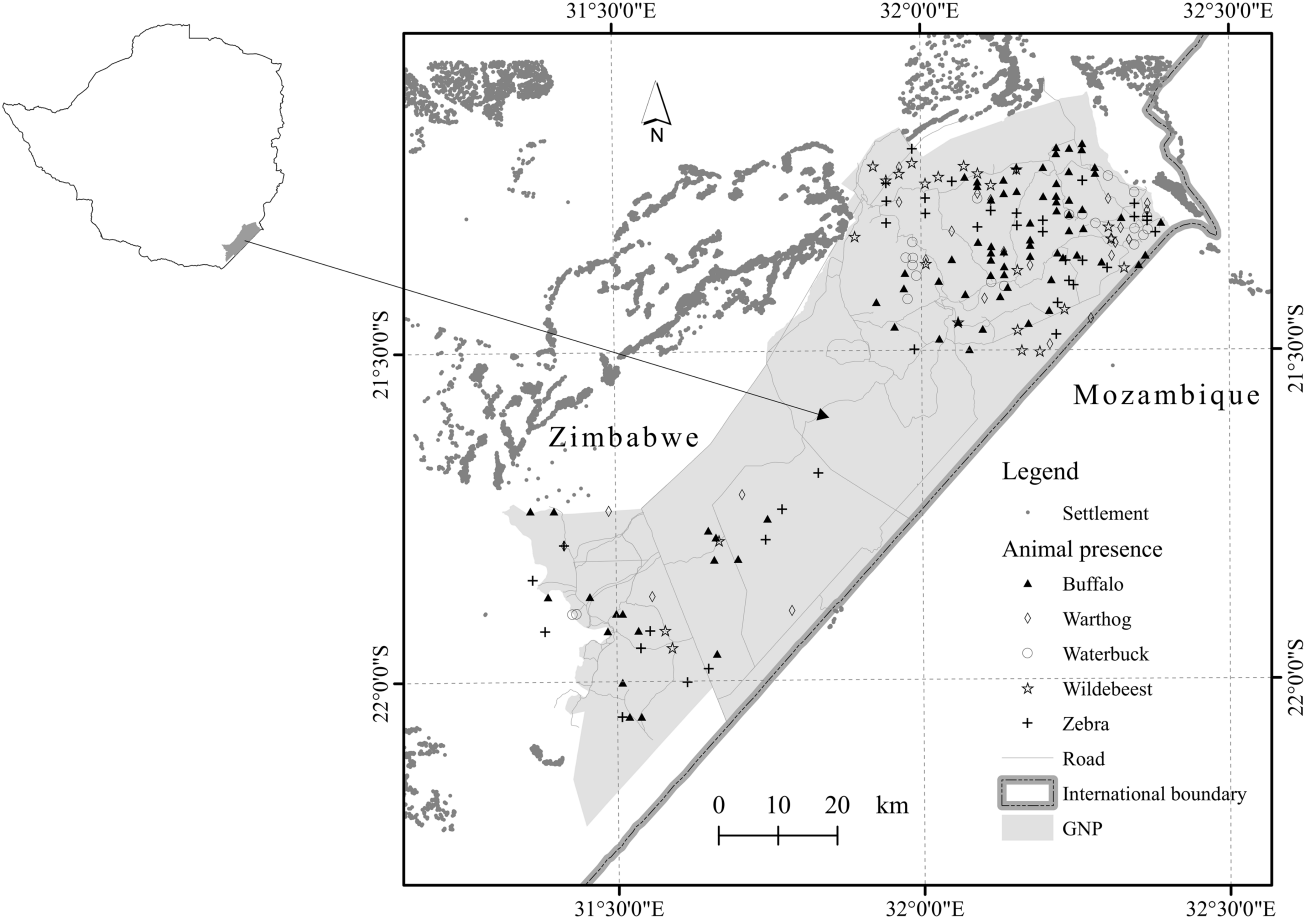
Location of the Gonarezhou National Park (GNP) in south-eastern Zimbabwe showing presence-only location data for five grazer species.

The study was conducted in the Gonarezhou National Park in south-eastern Zimbabwe (Fig 1). The park lies between longitudes 31.32°E– 32.41°E and between latitudes 21.11°S– 22.22°E. The park is the second largest in Zimbabwe with a considerable diversity of large mammalian species. The 2013 aerial survey estimated the population of our target grazer species as follows: buffalo (4425); warthog (484); waterbuck (578); wildebeest (1416); and, zebra (1685) [19]. Hunting and other forms of consumptive tourism are not permitted in the park and the main tourism activity is photographic safaris. Thus, the main sources of human interference with wildlife include tourist-carrying vehicles that drive along the main tourist routes as well as poaching for game meat from surrounding communities. Uncontrolled veld fires occur frequently in the park [20] and these are known to drive ungulate distribution in most ecosystems.

### Grazer presence data

Presence data for buffalo (n = 93), warthog (n = 27), waterbuck (n = 21), wildebeest (n = 24) and zebra (n = 44) were collected via an aerial survey conducted in the study area in October 2013. The aerial survey was a sample with transects spaced at 1.5 km and 2.5 km depending on animal density within sampling strata. A Cessna 185 fixed wing plane was used and two observers sitting at the right and left sides of the plane observed animals within strips of width 150 m. The observers searched for animals between two streamers fitted to the wing struts of the plane during pre-calibration flights in such a way that the search strip on the ground was 150 m wide. Each time animals were sighted, the location of the sighting was marked using a handheld GPS device (GPSmap 296, Garmin international). A full description of the method is provided in Norton-Griffiths [21].

### Environmental variables

Species distribution models were based on six predictor variables; Soil Adjusted Vegetation Index (SAVI), distance from roads, distance from settlements, distance from water, distance from the nearest animal sighting and fire frequency. Initially, we had seven environmental variables including short term fire scars but this was excluded in the final modelling since it exhibited evidence of multicollinearity. We tested for multicollinearity among the predictor variables using the Variance Inflation Factor (VIF) where the predictors with a VIF >10 were not included in the final modelling. The VIF was calculated using in Eq 1.

VIF=1(1−R2) (1)

We also tested for correlation between pairs of predictor variables using Pearson’s correlation coefficient (Table 1), and observed no multicollinearity amongst the predictor variables used to build the candidate models (|r|<0.7 [22]).

**Table 1:** Pearson correlation matrix for predictor variables.

	SAVI	Settlements	Roads	MNDWI
Distance from Settlements	0.204			
Distance from Roads	0.145	-0.036		
Distance from Water	0.014	0.134	0.091	
Fire Frequency	-0.086	-0.236	0.044	0.193
Distance from sighting other than Buffalo	0.050	-0.120	0.311	0.360
Distance from sighting other than Warthog	0.068	-0.096	0.343	0.320
Distance from sighting other than Waterbuck	0.052	-0.125	0.327	0.358
Distance from sighting other than Wildebeest	0.000	0.000	0.000	0.000
Distance from sighting other than Zebra	0.054	-0.124	0.339	0.360

SAVI is a proxy for grazing forage quantity which adjusts for bare ground [23]. The computation of SAVI was based on MODIS (MOD02QKM) images freely available at www.ladsweb.nascom.nasa.gov (accessed on December 12 2016). The MODIS images were acquired for October 2013, the same time when grazer presence data were collected. Pre-processing of the data included projection of the data from the sinusoidal (SIN) to the WGS 84 UTM Zone 36 South Coordinate Reference System to ensure compatibility with animal presence data described earlier. SAVI is a standard vegetation index calculated using Eq 2.

SAVI = ((NIR –R)/(NIR +R + 0.5))*(1 + 0.5) (2)

where NIR represents reflectance in the Near Infrared and R represents the Red band of the electromagnetic spectrum.

Distance from roads and distance from settlements were both used as indicators of human interference on wildlife e.g., through tourists conducting photographic safaris and illegal hunting. The roads and settlements were digitised on very high resolution images freely available on the Google Earth platform (www.googleearth.com). Later, distance from the roads and settlements were calculated using the Euclidean distance algorithm implemented in ArcGIS 10.1 [24].

We included distance from water as a predictor because drinking water is known to drive the distribution of most ungulates [25]. The active water points during the time of sampling were extracted from MODIS NDVI (MOD13Q1) data freely available on www.glovis.usgs.gov. The NDVI of water bodies is generally negative and the <0.0 threshold was used to classify water points following Huang et al [26]. We later calculated the distance of individual pixels from the nearest water point using the Euclidean distance calculation algorithm as described earlier.

The distance of individual pixels from the nearest recorded location of animal sighting was used as a proxy for interference competition where the pixels closer to the sighting represented elevated competition while those further away represented reduced competition. First, animal sighting data from the aerial survey described earlier were extracted. Second, the distance of individual pixels from the nearest location of an animal sighting other than the one whose distribution was being predicted was calculated using the Euclidian distance algorithm as described earlier. In this study, we used distance from the nearest sighting as an indirect measure of interference competition assuming that herbivores occupying the same space in the landscape tend to compete more for forage and other resources than those further apart.

Fire scars were used as a proxy for grazing forage quality where pixels with high fire frequency represented superior quality forage whilst those with low values represented inferior quality. Frequent fires are known to clear moribund vegetation and to promote the growth of fresh grass rich in nitrogen [27]. Fire scar data at the 500 m spatial resolution were obtained from the MODIS fire data platform made available via www.reverb.echo.nasa.gov (accessed on 1 December 2016). These data were downloaded for a 15 year period (2000–2014). Overlay analysis was later used to calculate fire frequency for individual pixels. Therefore fire frequency ranged from zero to 15 where zero represented pixels that were never burnt during the 15 year period while 15 represented those that were burnt each fire season of the 15 year period.

### Modelling approach

The Ensemble algorithm within the Biomode2 package in R [18] was used for modelling with the target species’ presence location data as the response variable and the six environmental variables described above as the predictors. Ensemble models were built, each for buffalo, warthog, waterbuck, wildebeest and zebra. For each SDM, 70% of the presence points were used to calibrate the model while the remaining 30% were used to validate the model. We used a threshold of ROC >0.6 for the Ensemble SDM following Thuiller et al [18].

The response of individual species to each of the six predictor variables was assessed using the response curves. For interpretation of the response curves, the logistic threshold of equal training sensitivity and specificity was used where the values above the threshold represented presence while those below represented absence. For overall model performance we used AUC following Panczykowski et al [28]. We also ran models without distance from the nearest animal sighting as a predictor to establish changes in model performance when the predictor was not used to build the model.

The contributions of individual predictors to the final model were tested using the variables importance calculation based on permutations implemented in the Biomod2 package in R. This statistic is based on calculation of the Pearson’s correlation between reference predictors and shuffled predictors. A predictor with a variable importance of zero has no influence on the final model.

## Results

The predictor variables i.e., SAVI as well as distance from roads, settlements and water, distance from the nearest sighting and fire frequency adequately explained the potential distribution of buffalo, waterbuck, wildebeest and zebra in the study area (AUC>0.90). However, the AUC for the model explaining the distribution of warthog was found to be 0.79.

Modelling without distance from the nearest sighting as a predictor variable reduced the AUC for buffalo (from 0.98 to 0.93), wildebeest (0.99 to 0.94) and zebra (from 0.97 to 0.93). However the AUC became better for warthog (from 0.79 to 0.94) and waterbuck (from 0.95 to 0.97) when the distance from the nearest sighting was not included as a predictor variable.

The variable importance analysis (Table 2) showed that distance from other animal sightings (used as a proxy for competition) was the best predictor of the potential distribution of buffalo (0.70), wildebeest (0.79) and zebra (0.79). However, distance from the nearest sighting was the second from most important variable in predicting the potential distribution of warthog (0.34) and waterbuck (0.12). Distance from settlements was the most important variable explaining the potential distribution of warthog whilst distance from water was the most important in explaining the distribution of waterbuck.

**Table 2:** Variable importance for each species distribution model.

Species	Variable	Variable importance
Buffalo	Distance from nearest sighting	0.701
	Distance from water (m)	0.052
	Distance from settlement (m)	0.046
	Fire frequency	0.035
	SAVI	0.023
	Distance from road (m)	0.004
Warthog	Distance from settlement (m)	0.699
	Distance from nearest sighting	0.336
	Fire frequency	0.013
	Distance from water (m)	0.002
	Distance from road (m)	0.001
	SAVI	0
Waterbuck	Distance from water (m)	0.767
	Distance from nearest sighting	0.116
	SAVI	0.032
	Fire frequency	0.03
	Distance from road (m)	0.024
	Distance from settlement (m)	0.017
Wildebeest	Distance from nearest sighting	0.789
	Distance from road (m)	0.082
	Distance from water (m)	0.049
	Distance from settlement (m)	0.03
	SAVI	0.02
	Fire frequency	0.015
Zebra	Distance from nearest sighting	0.777
	Distance from road (m)	0.068
	Distance from water (m)	0.047
	SAVI	0.045
	Distance from settlement (m)	0.035
	Fire frequency	0.007

**Fig 2:**
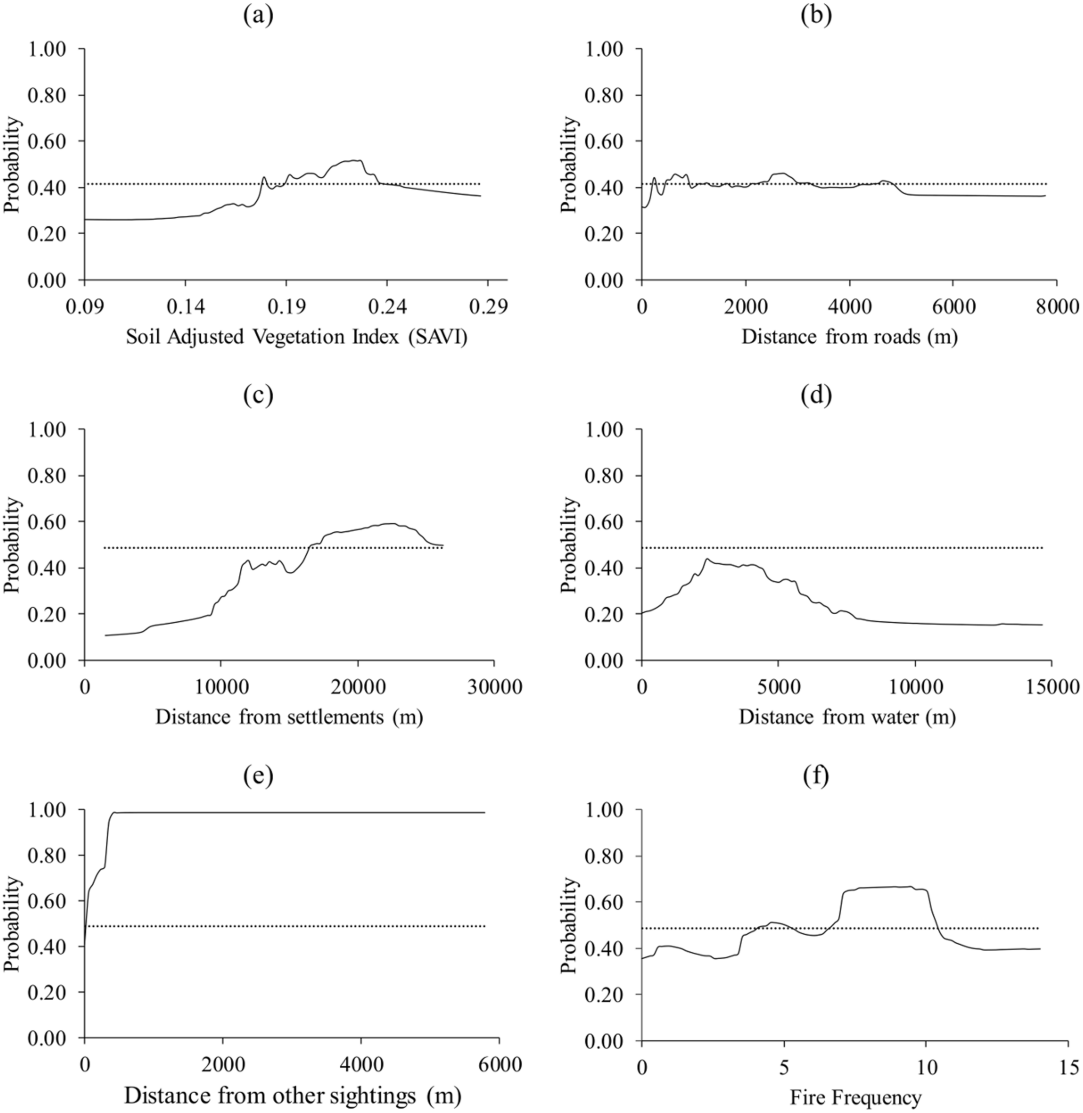
The response of buffalo to (a) Soil Adjusted Vegetation Index (SAVI), (b) distance from roads, (c) distance from settlements, (d) distance from water, (e) distance from nearest sighting and (f) fire frequency. Dotted lines represent the logistic threshold of equal training sensitivity and specificity.

Buffalo selected patches of medium SAVI (0.17–0.23), medium distance to roads (236–4875 m), medium distance to settlements (11–16 km), close to water (2372–3263 m), far from other sightings (>58 m) and of high fire frequency (4–11 fires in 15 years) (Fig 2).

**Fig 3:**
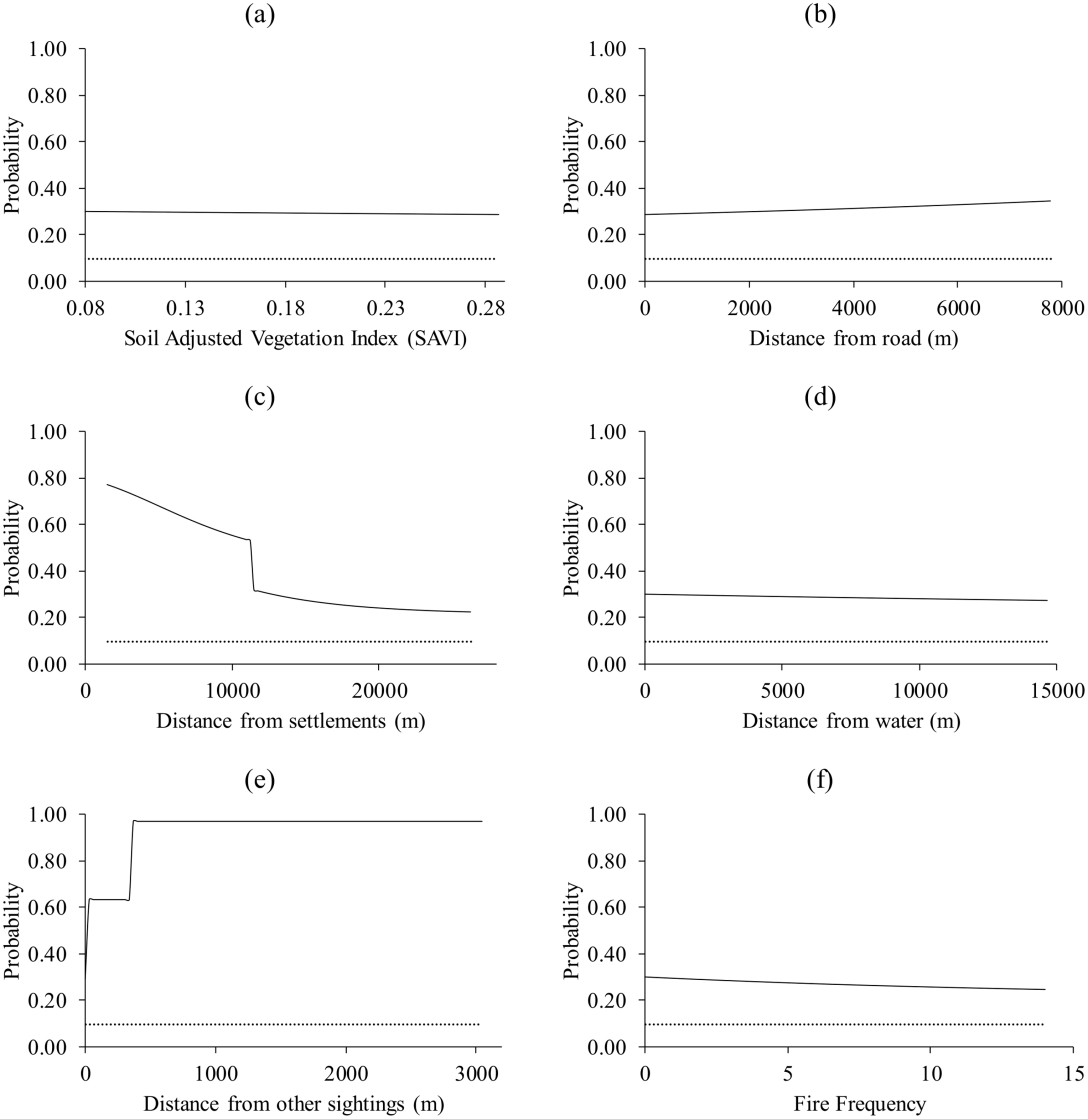
The response of warthog to (a) Soil Adjusted Vegetation Index (SAVI), (b) distance from roads, (c) distance from settlements, (d) distance from water, (e) distance from nearest sighting and (f) fire frequency. Dotted lines represent the logistic threshold of equal training sensitivity and specificity.

Response curves for warthog show that for all the six predictor variables, they selected patches with values above the logistic threshold of equal training sensitivity and specificity (Fig 3).

**Fig 4:**
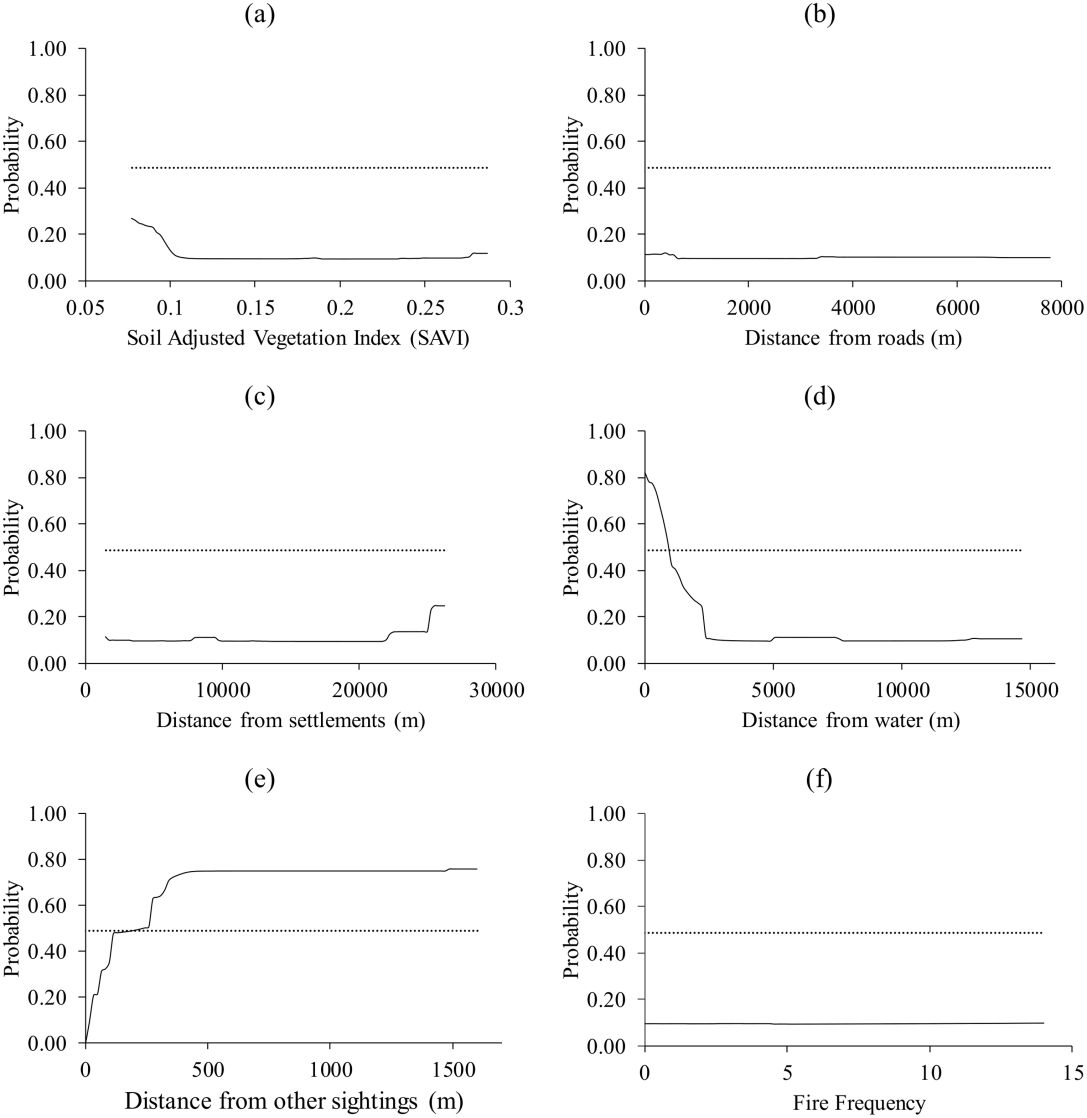
The response of waterbuck to (a) Soil Adjusted Vegetation Index (SAVI), (b) distance from roads, (c) distance from settlements, (d) distance from water, (e) distance from nearest sighting and (f) fire frequency. Dotted lines represent the logistic threshold of equal training sensitivity and specificity.

Waterbucks selected patches located far from other sightings (>163 m) and those close to water (<742 m) whilst the other four predictors were below the logistic threshold (Fig 4).

**Fig 5:**
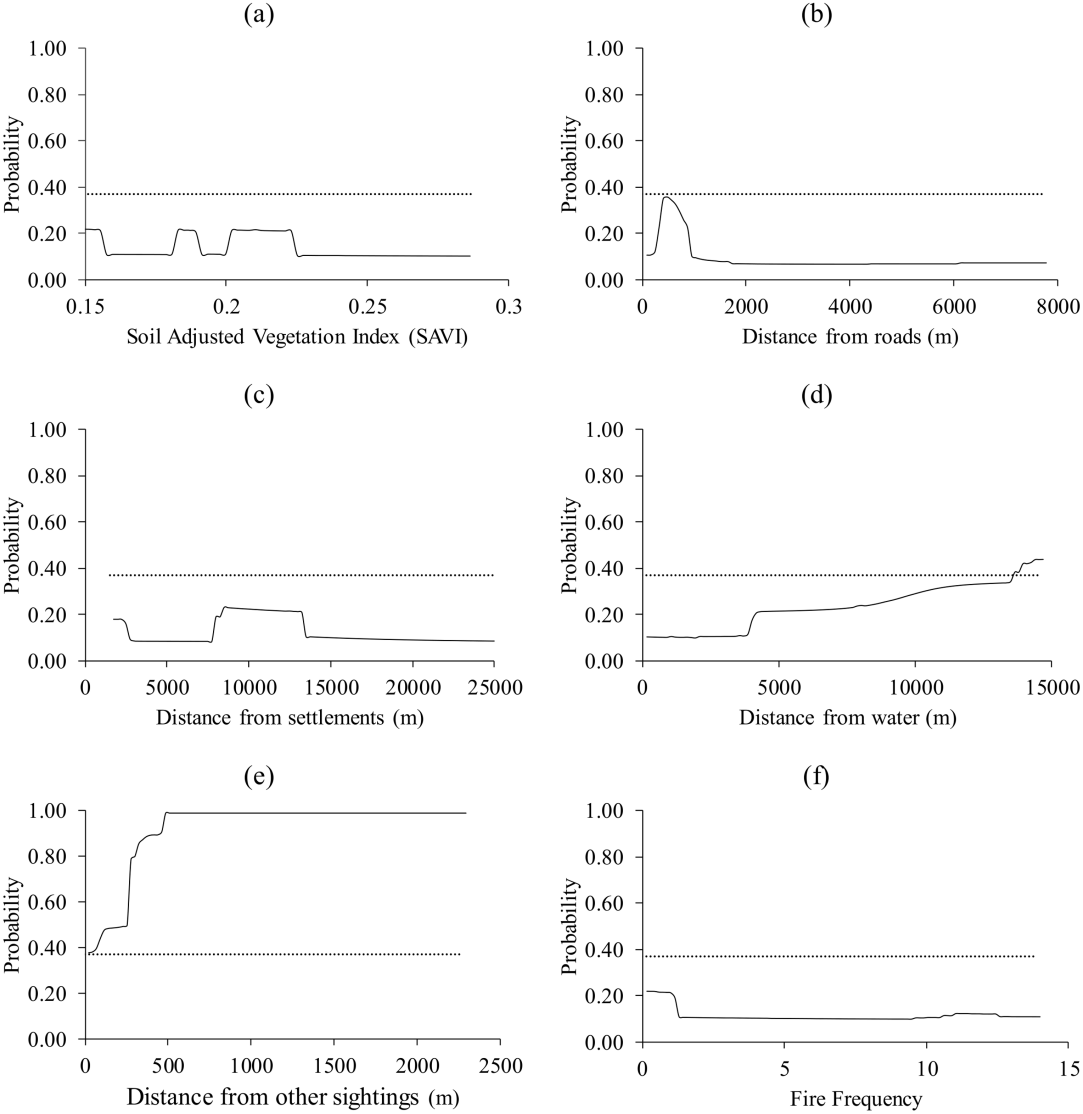
The response of wildebeest to (a) Soil Adjusted Vegetation Index (SAVI), (b) distance from roads, (c) distance from settlements, (d) distance from water, (e) distance from nearest sighting and (f) fire frequency. Dotted lines represent the logistic threshold of equal training sensitivity and specificity.

Wildebeest selected patches located far from water (>14 km) and also farther from other animal sightings but the other four predictor variables were below the logistic threshold (Fig 5).

**Fig 6:**
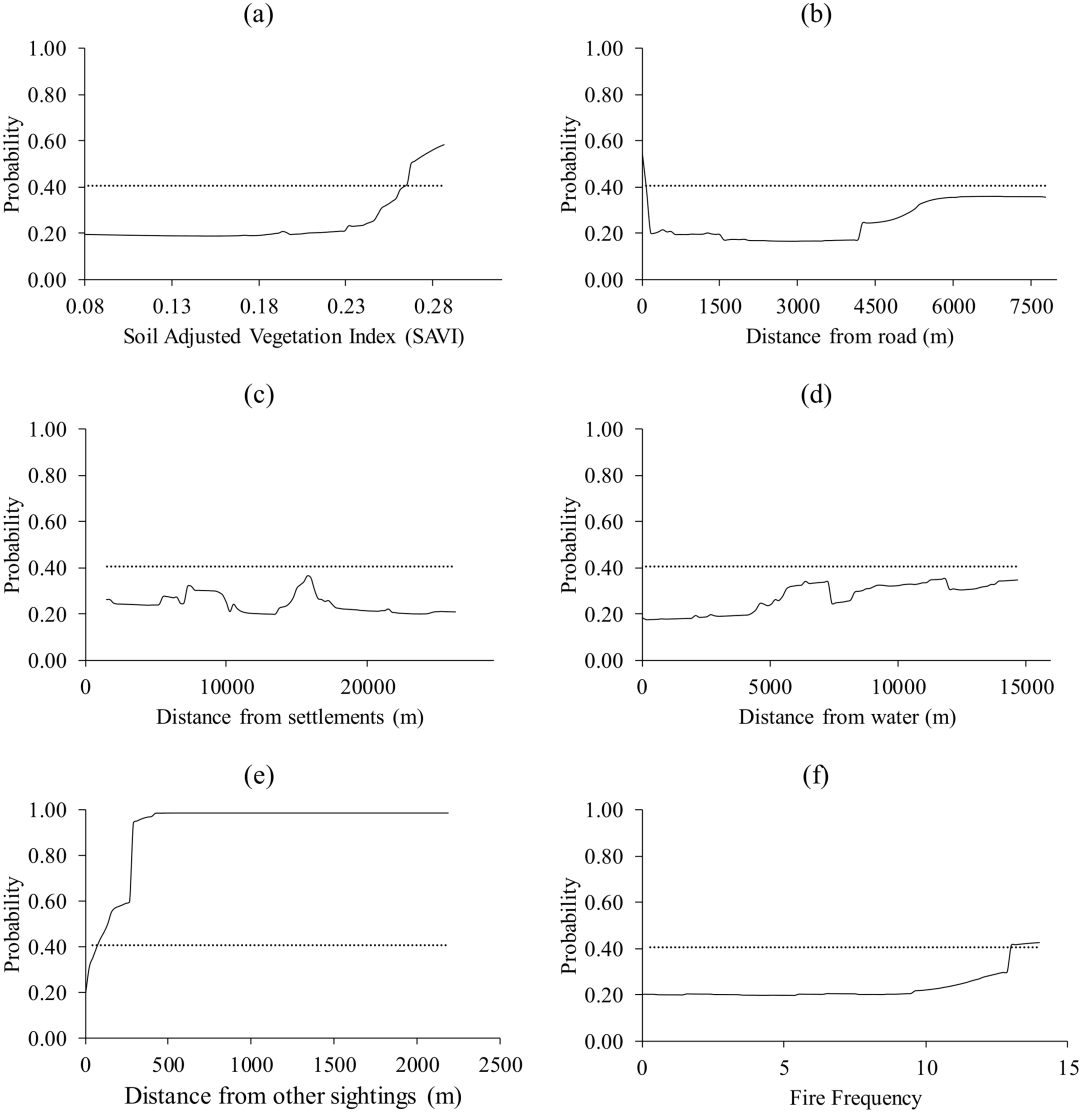
The response of zebra to (a) Soil Adjusted Vegetation Index (SAVI), (b) distance from roads, (c) distance from settlements, (d) distance from water, (e) distance from nearest sighting and (f) fire frequency. Dotted lines represent the logistic threshold of equal training sensitivity and specificity.

Zebras selected patches of high SAVI (>0.263), close to roads (<79 m), far from other sightings (>44.23 m) and also those patches of high fire frequency (>13 fires) whereas distance from settlements and distance from water were found to be below the logistic threshold (Fig 6).

## Discussion

We observed that interference competition (estimated by distance from the nearest animal sighting) explained the distribution of the target species better than forage (SAVI), interference from humans (distance from roads and settlements), distance from water and fire frequency. However, in tandem with past literature [29, 30], distance from water sources and distance from settlements were also important predictors of grazer distribution. SAVI was generally found to be the least important predictor.

These findings imply that using SDMs to predict the distribution of grazers without competition as a predictor may be inadequate. Our use of distance from the nearest sighting as a proxy for competition among grazer species for inclusion in SDM provides an important first step towards better characterisation of herbivore competition. Previous characterization of competition has largely been restricted to field based observations that are limited in both space and time [31, 32].

Results from the response curves consistently showed that all the target species avoided patches that are located near other animal species. In particular, peak probabilities of presence for the modelled species were observed at distances generally more than 500 m away from the next sighting. It could therefore be inferred that the patches located further away from other species are associated with less interference competition for resources such as forage and water. However, the effect of competition on the potential distribution of warthog and waterbuck was second best. For the warthog, distance from the nearest settlement was the most important predictor whilst for the waterbuck, distance from the nearest water source was the most important. While selection of patches close to settlements by warthogs is surprising, selection of patches close to water by the waterbuck could be explained by the fact that the antelope is largely water dependent [33, 34].

The major strength of our study lies in the inclusion of distance from the nearest sighting (a proxy for competition) with other traditional predictor variables to model the potential distribution of selected grazers. To the best of our knowledge competition is a missing variable in SDMs. Results from our study therefore provide impetus to explore newer approaches for characterizing competition in ecosystems. To date characterizing competition has been limited to point measurements. Our findings are also reliable because modelling was based on presence data from five different species, thus suggesting that the reported importance of competition is a consistent pattern rather than a product of chance. However, a possible limitation of our analyses is that, the way we estimated competition might not be the best since wildlife aerial surveys are sample estimates and therefore miss some animals in the landscape. In addition, results from our study give a snapshot of the predicted distribution of the study species during the time of sampling and as such cannot be easily used to represent their distribution at all times of the year.

## Conclusion

In this study we provided evidence for the importance of competition as a predictor for the geographic distribution of grazing ungulates. Our findings suggest that SDMs that exclude competition as a predictor variable might be inadequate in explaining the potential distribution of species in ecosystems. We therefore propose the inclusion of competition in SDMs.
